# Post-Donation Evaluation: Emotional Needs for Social Connection and Social Support among Living Kidney Donors—A Systematic Review

**DOI:** 10.3390/healthcare12121216

**Published:** 2024-06-18

**Authors:** Valentina Colonnello, Gaetano La Manna, Gabriella Cangini, Paolo Maria Russo

**Affiliations:** Department of Medical and Surgical Sciences, Alma Mater Studiorum, University of Bologna, 40138 Bologna, Italy; gaetano.lamanna@unibo.it (G.L.M.); gabriella.cangini2@unibo.it (G.C.);

**Keywords:** organ donation, prosocial, social support, nephrology, emotions, renal

## Abstract

Introduction: Evaluation of post-nephrectomy social health in living kidney donors is essential. This systematic review examines their emotional need for social relatedness post-donation. Methods: Following the PRISMA guidelines, we systematically searched Scopus, CINAHL, and PsycINFO. Results: Among the screened records, 32 quantitative and 16 qualitative papers met the inclusion criteria. Quantitative research predominantly utilized questionnaires featuring generic items on social functioning. However, a minority delved into emotional and social dimensions, aligning with qualitative studies emphasizing the importance of social connection and perceived social support post-donation. Specifically, post-donation changes in connecting with others encompass a sense of belongingness, heightened autonomy, shifts in concern for the recipient’s health, and continued care by shielding the recipient from personal health issues. Social acknowledgment and social support from both close and extended networks are reported as relevant for recovery after nephrectomy. Discussion: These findings underscore the necessity for targeted measures of emotional needs and social functioning to effectively assess post-donation adjustment. They also inform the identification of key health themes for kidney donor Patient-Reported Outcome Measures (PROMs) and Patient-Reported Experience Measures (PREMs) questions.

## 1. Introduction

A living kidney donation is considered a promising treatment option for patients with end-stage renal disease (ESRD). Donation itself is a social act aimed at preserving the bond and the health of an emotionally significant individual or, more broadly, an unspecified unrelated member of one’s own social group [1,2,3,4]. Therefore, it is important to understand the emotional and relational consequences of the donation of one’s own kidney.

While the living kidney transplant improves the recipient’s health, clinical practice guidelines require conducting follow-up evaluations for both the recipient and the donor after nephrectomy. The follow-up includes monitoring not only the physical but also the social health of the donors [5]. The social health dimension is grounded on the satisfaction of the basic emotional need of belonging, which needs to be considered one of the central areas of Patient-Reported Outcome Measures (PROMs) and Patient-Reported Experience Measures (PREMs).

As reviewed by Clemens and colleagues (2006), quantitative studies have reported mixed results on donors’ quality of life, ranging from no significant change to an improvement with respect to social relationships after donation. A minority of studies reported a negative impact on social relationships [6]. A more recent review by Liu and colleagues (2021) on the quality of life of living kidney donors found that social functioning remained unchanged [7]. Of note, the narrative review conducted by Hanson and Tong (2019) reported that, along with physical concerns, the impact on the family and the quality of the donor–recipient relationship are among the important outcomes from the donors’ perspective [8].

To the best of our knowledge, there is currently a gap in the literature when it comes to a systematic review of both quantitative and qualitative studies that specifically addresses the satisfaction of emotional need for social connection among living donors after nephrectomy. This review seeks, therefore, to cover this gap by focusing on how donors’ emotional need for social connection is assessed in quantitative studies post-donation and to what extent qualitative studies contribute to furthering the understanding of donors’ needs. Additionally, it explores differences in satisfaction with social bonding and connection between related and unspecified donors.

This study synthesizes existing quantitative and qualitative research, thus offering a clearer understanding of the emotional and social challenges encountered by living kidney donors and assessing the extent to which quantitative studies measure these needs. While quantitative studies provide valuable numerical insights into aspects such as satisfaction with quality of life post-donation, they may overlook the emotional dimensions experienced by donors, which hold crucial implications for policymakers. This review employs a mixed methods approach, harnessing the power of both narratives and numerical data [9].

The ultimate goal of this review is to enhance the identification of key health themes for kidney donor Patient-Reported Outcome Measures (PROMs) and Patient-Reported Experience Measures (PREMs) questions. These measures are crucial tools for monitoring the health status of living kidney donors and understanding their perception of healthcare services. Additionally, understanding donors’ emotional needs is crucial for supporting more individuals to consider living donation and supporting the healthcare system to foster a more positive donation experience.

## 2. Materials and Methods

### 2.1. Identification of Studies

The present systematic review was conducted in accordance with the Preferred Reporting Items for Systematic Reviews and Meta-Analyses (PRISMA) guidelines [10]. Through an electronic search in the Scopus, CINAHL, and PsycINFO databases, the following primary terms were used to search in the “title/abstract/keywords”: “renal donation” OR “kidney donor*” OR “renal donor*” OR “kidney donation” OR “living kidney donor*” AND TITLE-ABS-KEY (“social health” OR “quality of life” OR “health-related quality of life” OR psycholog* OR health OR psychiatry OR adjustment).

This search was limited to articles written in English and published in peer-reviewed journals between 1 January 2012 and 1 June 2023 (see Supplementary File S1 for a full description of the keywords used).

### 2.2. Eligibility Criteria

Articles were screened based on the following inclusion criteria: quantitative studies that included participants who donated a kidney and reported at least one measure of quality of life after donation; qualitative studies reporting on the post-donation experience.

Exclusion criteria were applied, which encompassed review, opinion, consensus, guideline, and single-case papers. Additionally, studies lacking information about the number of kidney donors or focusing solely on decision-making, expectations, and experiences before donation were excluded.

### 2.3. Screening and Data Extraction

After removing duplicate studies, the titles and abstracts of the remaining articles were examined to identify those that met the specified inclusion/exclusion criteria. Throughout this stage, the selection process prioritized sensitivity over specificity.

Hence, all articles that showed potential relevance were included in this study. In the subsequent step, the full text of all the included articles were identified through an initial electronic search. The screening and coding of the full articles were independently conducted by two reviewers (VC and PMR). Any discrepancies regarding the inclusion/exclusion process were resolved through discussions. The final selection of articles was based on the full-text examination.

For both the quantitative and qualitative studies, the data extraction stage involved collecting information on the author(s), publication year, country of origin, donor population details (including the number, age, and relationship to the recipients), and the time elapsed from donation to study participation. As for the quantitative studies only, the data coding form included details about the instrument used to assess social health and the findings related to satisfaction with social connection post-donation.

Concerning the qualitative records, key outcomes based on thematic analysis were recorded for each qualitative study. Initially, two independent authors (VC and PMR) analyzed the retrieved articles line by line to ensure a contextual interpretation. Subsequently, through a repeated reading process, these authors (VC and PMR) identified the descriptive themes. These themes were then examined for similarities, variations, and relationships, ultimately leading to the development of an analytical framework comprising higher-order themes through inductive coding [11].

## 3. Results

### 3.1. Summary of Included Studies

**Figure 1:**
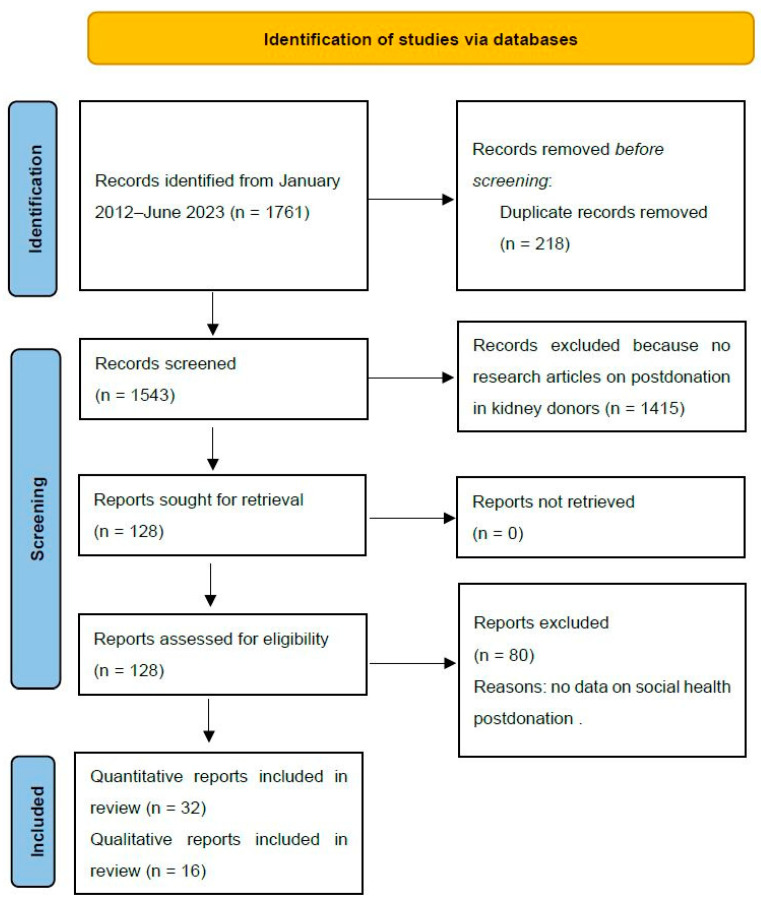
PRISMA flowchart and screening details.

The search strategy resulted in a total of 1761 articles. After eliminating duplicates (N = 218), the remaining articles underwent screening based on their titles and abstracts, leading to a selection of 127 articles for full-text assessment. Among these, a total of 48 articles (32 quantitative and 16 qualitative) were ultimately included. Please refer to Figure 1 for the PRISMA flowchart and screening details.

### 3.2. Quantitative Studies

Articles that involved the same population [2,12,13,14,15,16] were treated as a single study, resulting in a total of 28 studies with a cumulative sample size of *n* = 6712.

The studies encompassed a diverse range of countries, with research conducted in the United States [2,12,13,14,15,16,17], Germany [18,19,20,21,22], the Netherlands [23,24,25,26,27], India [1,28,29], Sweden [30,31], Brazil [32], China [33], Iran [34], Korea [35], Malaysia [36], Spain [37], Taiwan [38], Turkey [39,40], and the UK [41], and studies conducted across Canada and Australia [15,16]. Notably, most of the studies originated from Western countries.

Of the 28 studies, 13 included related donors (e.g., parents, siblings, partners, or friends) only [1,18,19,20,21,28,33,35,36,37,38,39,40], 12 studies included both related and unspecified donors [2,13,15,17,22,23,26,27,28,29,30,31,34,41], while 3 papers did not report the donor–recipient relationship [24,25,32]. The mean age of the donors ranged from 36.45 (SD = 16.9) to 58 (SD = 11) years. The age ranged from 20 to 83 years.

As reported in Table 1, 15 studies adopted a prospective design [13,14,15,16,19,20,21,24,25,26,27,28,29,31,32,37,41], and many of them collected data at multiple time points after donation. The post-donation observation period varied, encompassing a range from a few days to 3, 6 months, 1 year, and 2 years after donation in most of the studies.

As for the cross-sectional retrospective studies, they covered a wide timeframe, spanning from 1 to 38 years post-donation [1,12,17,18,22,23,30,33,34,36,38,39,40]. However, one study [35] did not provide information on the time elapsed from donation to study participation. In most of the cross-sectional studies, donors’ social functioning and relationships were compared with those of the general population [12,18,19,20,22,23,35,36,39].

As shown in Table 1, concerning the instruments utilized to measure post-donation social health, most of the studies employed the Short Form Health Survey [1,12,14,15,16,18,20,23,24,25,27,32,33,34,36,39] or the World Health Organization Quality of Life (WHOQoL) BREF [19,21,28,29], regardless of the country of origin. Several studies incorporated ad hoc-developed questionnaires [1,2,30,40]. These questionnaires’ items were related to the effects of donation on family relationships or the individual’s social network, but comprehensive information about their reliability and validation was not always reported.

Among the prospective studies, 10 reported no changes in social functioning post-donation compared to pre-donation [14,15,20,21,24,27,29,31,37,41]. Four studies indicated a decline in social functioning post-donation [16,19,27,32], while three studies reported improved social health or stronger relationships post-donation compared to baseline pre-donation [13,25,28]. Both studies reporting a decline and an improvement in social health were based on data collected during the first two years post-donation.

Regarding the retrospective studies, three studies found that donors’ social functioning was better than that of the general population [22,23,36] and that donation had a positive influence on their relationship with the recipient [1,2] and recognition from the society [1]. Lower social support was associated with lower positive effect post-donation [38], and social support and satisfaction of the relatedness need were associated with donors’ post-traumatic growth [35]. By contrast, three studies observed no significant differences between donors’ and the general population’s social functioning. Among the six studies based on data collected over five years after donation [1,2,12,18,22,23,38], four studies [1,2,12,22,23] reported an improvement in social functioning.

Regarding the question of whether there are differences in social health between related and unspecified donors, one study reported that paid unrelated donors had lower social functioning compared to related donors [34], while another study reported that spouse donors and non-spouse donors do not differ in marriage satisfaction [17]. However, no other studies explicitly investigated differences between related and unspecified donors. Nevertheless, a study involving related donors and comparing subpopulations of donors reported that donors’ post-donation experiences were related to the type of relationship with the recipients [38].

**Table 1:** Summary of articles based on quantitative studies.

Social Health Change	Instrument	N of Donors	Age in Years Mean (SD)	Relationship with Recipient	Study’s Timeframe	Reference
Stronger relationship with recipients; feelings of recognition in society	SF-36; ad hoc questionnaire	506	37.99 (N.R.)	Primary social group	over 9 years post-donation	[1]
Better relationship with the recipient. Better social functioning than the general population.	Ad hoc questionnaire; SF-36	2455 2455	58 (11) 58 (11)	Primary social group, unspecified primary social group, and unspecified	17 years post-donation 17 years post-donation	[2]
						[12]
Improved interpersonal benefits. No changes in social functioning for the majority of donors.	LDEQ SF-36	133 123	43.10 (11.2) 44.10 (11.2)	Primary social group, unspecified primary social group, and unspecified	Pre-donation, and 1 and 6 months post-donation, and 1 year and 2 years post-donation; Pre-donation, 1 year post-donation, and 2 years post-donation	[13]
						[14]
Non-clinically significant decline in social functioning. Worsening of social functioning at 3 months post-donation.	SF-36 SF-36	912 821	48 (N.R.) 49 (N.R.)	Primary social group, unspecified primary social group, and unspecified	Pre-donation, 3 months post-donation, and 1 year post-donation Pre-donation, 3 months post-donation, and 1 year post-donation	[15]
						[16]
Spouse donors and non-spouse donors do not differ in quality of marriage scores	Revised Dyadic Adjustment Scale	42	52.40 (10.5)	Primary social group and unspecified	Post-donation. Years not reported	[17]
No difference in social functioning than the general population	SF-36	55	49.20 (10.10)	Primary social group	6.2 years post-donation	[18]
Decline in quality of life at 3 months post-donation, but it was comparable to the general population.	WHOQoL-BREF	41	50.72 (10.34)	Primary social group and no data	Pre-donation and 3 months post-donation	[19]
No social functioning changes across time; social functioning decreased compared to the general population at 3 months post-donation; the donor’s mental health moderately correlated with the recipient’s health.	SF-36	58	54.30 (11.7)	Primary social group	Pre-donation, 3 months post-donation, and 1 year post-donation	[20]
No clinically significant changes in social relationship post-donation.	WHOQoL-BREF	50	55.0 (11.1)	Primary social group	Pre-donation and 3 months post-donation.	[21]
Unchanged or improved relationship to the recipient. Better quality of marriage than general population.	Quality of Marriage Index	361	57.20 (9.3)	Primary social group and unspecified	Post-donation: 1–38 years	[22]
Better social functioning than the general population	SF; ad hoc questions on social relationship changes	316	51.70 (11.4)	Primary social group and unspecified	5.07 years post-donation	[23]
No changes in social functioning	SF-36	74	49 (N.R.)	Not reported	Pre-donation and 10 years post-donation.	[24]
Improvement of social functioning post-donation	SF-36	23	54.90 (13.30)	Not reported	Pre-donation and 3 months post-donation.	[25]
Lower social support was related to lower positive effect at all time points	SSL-I; SSL-D; LDEQ	135	55 (N.R.)	Primary social group and unspecified	Pre-donation, 3 months post-donation, and 1 year post-donation	[26]
Worsening in social functioning at 6 months and 1 year post-donation; quality of the donor–recipient relationship did not change over time; the donor’s life was less influenced by the recipient’s health	RAND-SF36 and ad hoc developed Perceived Donation Consequences Scale	230	55.10 (10.70)	Primary social group and unspecified	Pre-donation, 6 months post-donation, and 1 year post-donation	[27]
Improved social relationship at 3 months post-donation	WHOQoL-BREF	30	43.77 (10.64)	Primary social group	Pre-donation, 2 weeks post-donation, and 3 months post-donation.	[28]
No changes in social relationships at 3 months post-donation	WHOQoL-BREF	39	41.74 (8.85)	Primary group and unspecified	Pre-donation and 3 months post-donation.	[29]
Wanting but not having been offered a mentor is reported as early predictor for less favorable outcomes post-donation.	Ad hoc questionnaire	171	N.R. (N.R.)	Primary social group and unspecified	1–7 years post-donation	[30]
No changes in social activities and social support at any post-donation times.	Dartmouth COOP Functional Health Assessment Chart	112	50 (N.R.)	Primary social group and unspecified	Pre-donation, 3–4 weeks post-donation, and 6 months post-donation	[31]
Worsening in social functioning 1 month postdonation.	SF-36	110	42.20 (9.45)	Not reported	Pre-donation and 1 month post-donation.	[32]
Social functioning and social support for sibling donors was better than those of parent donors	SSRS; SF-36	98	49.20 (6.90)	Primary social group	2 years post-donation	[33]
Worse social functioning in paid unrelated donors than related donors	SF-36	144	36.45 (16.90)	Primary social group and unspecified	3.4 years post-donation	[34]
Relatedness of self-determination and social support were positively related to donors’ post-traumatic growth. No comparison pre/post-donation or to the general population.	MSPSS;BPNI	114	54.40 (10.1)	Primary social group	N.R.	[35]
Better social functioning than the general population	SF-36	80	N.R. (N.R.)	Primary social group	about 1–20 years post-donation	[36]
No changes in social functioning at 1 year post-donation	SF-36	60	50.20 (11.70)	Primary social group	Pre-donation and 1 year post-donation.	[37]
Sibling donors reported greater negative affect than donors who were the children of or in a couple with the recipients	SF-36 PANAS	41	49.79 (11.46)	Primary social group	5 years post-donation	[38]
No changes in social functioning than the general population	SF-36	36	42.0 (10.90)	Primary social group	1–2 years post-donation	[39]
No changes or improvement in social relationship with recipient post-donation	Ad hoc questions	208	48.74 (11.78)	Primary social group	4.55 years Post-donation	[40]
No changes in social comparison or social support at 3 months and 1 year post-donation.	Office of National Statistics Wellbeing questions; MSSS; social comparison	93	45.0 (12.98)	Primary social group and unspecified	Pre-donation, 3 months post-donation, and 1 year post-donation.	[41]

SF, Short Form Health Survey; WHOQoL-BREF, World Health Organization Quality of Life-BREF; PANAS, Positive and Negative Affective Schedule; SSL-I, Social Support List-Interaction; SSL-D, Social Support List-Discrepancies; LDEQ, Living Donation Expectancies Questionnaire; MSPSS, Multidimensional Scale of Perceived Social Support, and BPNI, Basic Psychological Needs Index; SSRS, Social Support Rating Scale. The donors’ age is reported as the mean in years with the corresponding standard deviation (SD) where available. For data not reported (N.R.), this is indicated accordingly.

### 3.3. Qualitative Studies

The selected studies originated from various countries, including the United States [42,43,44,45,46,47], the UK [3,48], Denmark [49,50], the Netherlands [51], Japan [52], Canada [53], Australia [4], and across Canada and Australia [54,55]. Thus, most of the research comes from Western countries.

The articles by Halverson and colleagues [42,43], as well as the articles by Agerskov and colleagues [49,50], were considered as two single studies each since they referred to the same population.

The combined sample size of the selected studies was 573 participants. Based on the studies that reported the mean age of the donors, the donors’ ages ranged from 21 to 89 years at the time of donation, with a mean age between 44 and 50 years. There were no significant differences in donor ages among the countries represented. Similar to the quantitative studies, the time elapsed between donation and study participation ranged from 2 weeks to 35 years. The data were collected using semi-structured interviews and focus groups.

As presented in Table 2, the thematic analysis of the papers resulted in the identification of two main themes concerning the perception of social health: “connection to others” and “social support”. The first theme explores how the act of donation influenced individuals’ perceptions and expectations regarding their connection to others, while the second theme delves into the impact of social support on the post-donation recovery process. These main themes include specific descriptive subthemes.

It is important to note that no direct comparison between unspecified and related donors was reported in any of the articles. However, some subthemes were specifically attributed to related donors (e.g., apprehension toward the recipient’s health or shielding the recipient).

#### 3.3.1. Connection to Others

This theme encompasses several subthemes related to the effects of donation on social interactions and dynamics. These subthemes include feelings of belonging, dependence reduction with an increase in autonomy feelings, apprehension about the recipient’s health, and shielding the recipient. The titles of these subthemes highlight the dominant emotions associated with social health post-donation.

1. Sense of Belongingness.

This subtheme focuses on donors’ perceptions of changes in their social connections and networks following a donation. These changes may involve the strengthening of existing relationships and the establishment of new connections. The sense of belongingness can be expressed as a feeling of being an integral part of a system and a community that is meant to grow and thrive. For example, an unspecified donor shared the thought that *donation “it is a big life thing, so I like the idea that you remain part of a system”* [4]. Additionally, another donor reported the following:

“*I shared a story about my kidney donation of Facebook, to spread the word about living kidney donation*”.

[51]

For certain donors, donations appear to have facilitated the deepening of existing relationships:

“*In all the ways really, I mean all the relationships I have, have been made better by this experience. I think the reason it was me because people talk to me differently, people spoke to me more emotionally and more honestly because of the experience…it was like it was inviting them in and I think once you’ve done that it’s continuous, the benefit just continues to grow*”, as expressed by some unspecified donors.

[3]

Regarding changes within family relationships, some related donors perceived that the act of donation “united” the family [44] and strengthened family ties, fostering feelings of sharing and belonging, resulting in a sense of “being a part of each other” [49]. In the case of spouse donors, some perceived that donation strengthened their marriage [48].

Donors may also experience a need to prevent donation from adding ambiguity or disrupting reciprocity in their relationships:

“*It belongs to her, I remember this, I wrote her a letter, I told her, it belongs to you, no longer to me, you can do whatever you want with it*”.

[53]

2. Autonomy feelings.

This subtheme was identified specifically for related donors, and it pertains to the positive effects of donation on donors’ autonomy. For related donors, the restoration of the recipient’s health following the donation led to an enhancement of the donors’ autonomy in their daily life and family activities [47,48,52,55]. Hanson [55] quoted a woman from Canada in her 50s who said, “Ever since then we have been traveling all over the world. So freedom! The freedom is amazing, so that is really important to us”. Takada [52] mentioned a participant who said:

“*The recipient could go back to work and could go to work alone without me escorting him, which made me feel that the transplantation was a great help”,* while Rasmussen [47] reported: “*Tonight I’m going to have to work late. I mean, we’re able to do that because I don’t have to rush home to put him on a machine*”.

3. Apprehension for recipients’ health.

The subtheme “Apprehension for recipients’ health” specifically addresses the effects of donation on changes in donors’ apprehension regarding the recipients’ health. Donors’ experiences of donation are closely connected to the recovery of the recipients. As a result, while most donors declared that the restoring of recipients’ health was a “relief” and helped to overcome one’s own post-donation health issues, some donors expressed concerns for the well-being of the recipients [45,49,50,52], and certain donors found the possibility of complications during the recipients’ recovery to be emotionally challenging. For example, as stated by Hanson [55], a woman from Canada in her 50s shared her thoughts:

“*If my husband’s kidney failed, I’m not sure how I’d react to that. Right now he’s doing extremely well. But if something should happen to him, would I be exposed to this depression and anxiety? That would worry me*”.

4. Shielding the recipient.

Donation affects the dynamics between the donor and the recipient and influences the sharing of mutual vulnerability. The act of transplantation challenges the donors’ health conditions, and some related donors wish to shield the recipients from potential problems [43,49,55].

After undergoing a nephrectomy, one participant shared her experience:

“*Even now I still don’t get any feeling in my arm when I wake up. It is disturbing. I don’t make a big deal of it because I donated my kidney to my sister, and I don’t want her to think that my lifestyle has changed*”.

[55]

Moreover, some donors who themselves developed kidney disease felt the need to conceal their health issue to protect the recipients from negative feelings. As described in [43], a donor with kidney disease reported:

“*I hesitated… waited [sic] to tell my mom because I didn’t want her to feel guilty*”.

#### 3.3.2. Social Support

The second theme identified is related to “social support”. This theme highlights the importance of social acknowledgment from both family members and the wider community. Donors may seek acknowledgment and social support from their primary social group (family and close friends), healthcare providers, and extended network.

1. Need to be recognized and acknowledged for the efforts.

This subtheme emphasizes the fact that a crucial aspect of donors’ satisfaction with social health post-donation is dependent on how donors perceive the acknowledgment of their perspective by recipients and healthcare providers.

Donors expressed that their act of donation was a “natural” action, and they were not seeking a “hero status” [55]. However, they also felt that their actions should not be underestimated, neither by healthcare providers nor by recipients.

For example, a donor shared: “[The doctor] just very simply said, “thank you for the kidney” I so hadn’t expect it. It just blew me away” [4]. In addition, a donor stated: “There was a sense of entitlement from some recipients, their physicians, their teams. There was a thought that you’re like taking a medication off the shelf” [45].

As a donor mentioned:

“*I’ve always found it a shame that I did not hear anything. How on earth it is possible that someone receives a kidney and does not even send a postcard or a soap bar or just something, a gesture, I do not understand it*”.

[51]

Another donor stated:

“*I was slightly concerned [about] what sort of person who would receive a kidney and then ignore the donor? So I think sending a thank you at least is probably… I mean I wouldn’t expect much but sending a thank you seems like common decency*”.

[3]

For some related donors, recipients’ noncompliance with medical recommendations was perceived as a sign of poor attention to the received kidney. For instance, a donor shared:

“*Just recently things have not gone that well for my husband even though the transplant was initially successful. The reason why is my husband has continued to smoke… This is a hard issue for me*” [44]. Similarly, in [49], a donor expressed disappointment that the recipient had put on weight, stating: “*I was disappointed that he [the recipient] had put on weightb… I thought: ’Ok, you haven’t been looking after yourself properly*”.

On the other hand, some donors reported that the interpersonal benefits of donation included receiving appreciation and respect from both their immediate family and their extended social network. As another donor reported:

“*I think it just made him [the recipient] appreciate the sacrifice I was willing to make a little more. I think it made him respect me and appreciate me a little bit more than he did previously*”.

Additionally, a donor reported:

“*As far as elevating the status, like the people that are aware of what we’ve been through … from the church and the community, they put us you know, they looked at us in a different way. So, we enjoyed some privileges*”.

[48]

2. Support from the Primary Social Group.

Donors reported that their recovery from nephrectomy was eased by the support they received from members of their primary social group, including relatives and close friends. This emphasizes the importance of social support in the donors’ reported recovery experience [42,43,49].

For instance, one donor described the pain after surgery and the support they received:

“*I’ve had really good social support like more than what I would have thought…the only thing that’s been like… harder than I thought was the pain*”.

[46]

Conversely, the lack of support was perceived as conflicting with recovery. One donor advised:

“*Make sure that donors have a good support group around them…at least a couple of people that would drop in and see if they’re okay”. Some donors described discomfort due to the “lack of support from family members*”.

[55]

Social support from family members was not without tension. As stated by a donor:

“*After the donation, I stayed with my sister for 6 weeks. Everyone wanted to help me, but they also do that when I have a normal flu*”.

[51]

3. Healthcare providers’ support.

This subtheme emerged both during the immediate post-donation experience/period and during the follow-up [4,50,55]. Donors appreciated that the support from healthcare providers was not limited to physical conditions but also included “a lot about mental wellbeing” [50]. Donors reported both positive and negative experiences of support received from healthcare providers. In particular, healthcare providers’ support let some donors experience “a sense that they’re looking out for the person” [54], while some donors complained about the “relatively little follow-up and discussion after things… and it was very test-related” [54].

The complaints were related to the reduced attention received from healthcare providers after the donation. Some donors reported being disappointed by the perceived gap between the attention and care received before the donation and the attention and care post-donation. For example, one donor complained that the care received was:

“*Virtually nil. I came out in early July and was seen at the end of August, and apart from that, there’s nothing now. It will be a year before I see anybody again. That’s disconcerting; it’s almost thanks and good-bye*”.

[47]

Similarly, another donor noted that:

“*After you make the donation of your kidney, nobody from that hospital where you donated said, ‘Hey, come back in here so we can check you’re doing okay*”.

[45]

4. Extended social group support.

Social health is dependent on the broad social network, including interactions in the work context. One of the subthemes that emerged from the analysis is that the understanding and acknowledgment in the work context for the decision to donate plays a key role in determining the experience post-donation. For example, a donor stated that:

“*At work, they did not cooperate at all. I work in the healthcare sector, but they were not supportive… It made me very sad*”.

[51]

Another donor reported:

“*I was actually fired for the amount of time that I was about to take off… apart from the actual firing itself, I found it very hard after the recovery of about 2 months to get another job. People just didn’t want to look at me. It was the weirdest thing ever*”.

[55]

Support sometimes came from the donors’ blogs and online resources, which allowed the donors to appraise their own experiences, considering the experiences of an extended virtual network of donors. As stated in [55]:

“*And that is why the donor blogs were super helpful because there are some people on there who have had truly awful experiences. And I knew that wasn’t going to be my experience. There were some people on there who had truly amazing experiences, and mine ended up somewhere in the middle*”.

## 4. Discussion and Conclusions

This systematic review sheds light on donors’ emotional needs after nephrectomy. Unlike previous reviews that focused solely on quantitative or qualitative studies, this review provides a comprehensive synthesis of both types of studies to facilitate the identification of the most useful themes for Patient-Reported Outcome Measures (PROMs) and Patient-Reported Experience Measures (PREMs) in the population of kidney donors.

Our findings indicate that most of the quantitative studies used questionnaires that limitedly explored donors’ social experience post-donation. These questionnaires, such as the Short Form Health Survey and the WHOQoL-BREF, typically included only one to three generic items related to social functioning. These studies reported mixed results on donors’ social health, particularly when assessing the first couple of years after donation. However, a consistency has been found in retrospective studies based on data collected over five years after donation, with most reporting an improvement in social functioning.

This suggests that donation may have a positive long-term impact or that, over time, donors maintain a positive evaluation of the donation experience.

This tendency to use generic questionnaires in the kidney donor population and the mixed results is consistent with findings from other studies, such as the review conducted by Clemens and colleagues in 2006 [6], which also highlighted the prevalent use of the Short Form Health Survey. We found that, since then, the use of generic questionnaires remained, which may dramatically limit the understanding of donors’ emotional experience after nephrectomy.

The limited number of quantitative studies that expanded the assessment of social health post-donation utilized ad hoc-developed questionnaires [1,2,23,30], often lacking comprehensive information on the validity and reliability of their instruments. Despite these limitations, these studies did draw attention to crucial aspects of donors’ social and emotional health, such as the support received during the donation process [23,30]. Notably, there are studies that stand out as an exception, as they delve into donors’ emotional experiences using validated questionnaires. These studies underscore the importance of evaluating satisfaction with social support, as well as the fulfillment of the relatedness need [22,33,35]. These findings are consistent with the conclusions drawn from the qualitative studies reviewed in this analysis. Specifically, our review has identified two primary themes that warrant further exploration in future quantitative research.

The first theme, “connecting to others”, encompasses several subthemes that shed light on the effects of donation on the donor’s social interactions and expectations. This includes the increased feeling of belongingness to a community or a family unit, as the act of donation can create a stronger bond between the donor and the recipient or to a community. The improved health of the recipient following the donation can also lead to an increased sense of autonomy for the donor. However, there may be apprehension and concern about the recipient’s health, with whom the donor shares a bond, leading to fears of burdening the recipient by revealing any personal health issues that may arise as a consequence of the donation.

The second theme, “social support”, emphasizes donors’ need for social acknowledgment from family members and the broader community following kidney donation. Donors may seek acknowledgment and understanding from their immediate family members and social network due to the significant impact that the act of donation can have on various aspects of their lives. However, this theme also reveals potential sources of disappointment or challenges that donors may encounter in relation to the social support they received. The theme of “social support” includes subthemes that delve into the effects of support from various sources, such as family members, healthcare providers, and the extended social network. This highlights that donors’ post-donation social health is intricately intertwined with their perception of the support they receive from different individuals and social groups.

One of the research questions underlying this review was on whether there are differences in social health between related and unspecified donors. Although several of the qualitative and quantitative studies included both related and unspecified donors, they did not provide separate data for the two subpopulations, which makes it challenging to draw definitive conclusions on the potential differences in social health between these groups. The few papers that considered the related and unrelated donors’ perspectives suggested that the nature of the donor–recipient relationship may play a role in post-donation social health outcomes. Future research needs to investigate possible differences between related and unspecified donors during post-donation adjustment. Specifically, for donors who also serve as caregivers for the recipients, it is essential to adopt a research approach that considers the adjustment of the couple to the donation, such as the actor–partner interdependence model [56]. Additionally, future research should compare outcomes and emotional needs for social connection and support between direct-related living donors and those involved in living donor-paired exchange programs.

Of note, the majority of both quantitative and qualitative studies originate from Western countries, yet no significant differences in donor ages were observed among the represented countries. Quantitative studies utilized standardized quality of life questionnaires across various countries, while qualitative studies consistently identified themes regardless of their country of origin. This finding suggests a certain level of consistency across different cultural contexts, despite variations in the incidence of living kidney donation across countries [57].

This review has several limitations. Firstly, despite an initial broad search of terms to minimize publication bias, it is possible that some relevant papers might have been inadvertently excluded. Additionally, the selected papers often relied on retrospective studies, where participants were asked to recall information about their experiences after varying lengths of time since their donation, ranging from a few days to nearly 40 years. As a result, donors’ perspectives and findings, and consequently, the conclusions of this review, may have been influenced by a post-hoc reappraisal of donors’ experiences. Furthermore, the absence of analysis on gender differences in donors’ emotional needs in the majority of studies hinders the ability to discern such variations. Future studies should prioritize addressing this aspect, particularly given the predominance of women as donors [58].

Despite these limitations, the findings of this review contribute significantly to the identification of central themes crucial for the development of Patient-Reported Outcome Measures (PROMs) and Patient-Reported Experience Measures (PREMs) related to social health among living kidney donors. With respect to PROMs, this review suggests that the key issues and concerns important for the social health domain include satisfaction of emotional need for meaningful connections with others, ranging from feelings of belongingness to autonomy-dependence regulation, apprehension about the recipients’ health, and continued care for recipients. These findings underscore the necessity of integrating more focused measures of social functioning into current post-donation evaluation screening protocols.

As for PREMs, the themes deemed crucial for measuring good quality of care include providing donors with opportunities for social support from closer and extended networks. In line with the literature on the role of positive social interactions in health [59], this review emphasizes that satisfaction with the emotional need of being supported and socially acknowledged can play a role in post-donation experience.

While further research is required, the findings of this review highlight the limitations of current quantitative studies focusing on general aspect of social functioning and the importance of integrating quantitative and qualitative studies to gather insights into donors’ emotional needs. These insights can be utilized by scholars to further the selection and development of measures to assess donors’ health and by healthcare providers and support networks to better assist and guide kidney donors throughout their post-donation emotional adjustment.

## Figures and Tables

**Table 2:** Themes, subthemes, and supporting studies.

Theme	Descriptive Subtheme	Supporting Studies
1. Connection to others	Belonginess feeling	[3,4,47,48,49,50,51,53]
	Autonomy feelings	[47,48,52]
	Apprehension for recipients’ health	[44,47,49,50,52,55]
	Shielding the recipient	[43,49,55]
2. Social support	Need to be recognized and acknowledged	[3,44,46,48,49,51,54]
	Support from primary social group	[42,46,51,54]
	Healthcare providers’ support	[4,42,45,47,49,51,54,55]
	Support from extended network	[46,51]

## Data Availability

Data supporting this systematic review are available in the cited publications.

## Supplementary Materials

The following supporting information can be downloaded at: https://www.mdpi.com/article/10.3390/healthcare12121216/s1, Supplementary File S1: Keywords used for article search.
