# Supplementary material for: Post-Donation Evaluation: Emotional Needs for Social Connection and Social Support among Living Kidney Donors—A Systematic Review
Source: Healthcare (Basel). 2024 Jun 18;12(12):1216. doi: 10.3390/healthcare12121216 (PMC11203999; doi:10.3390/healthcare12121216)
Supplement: Supplementary file 1 [file healthcare-12-01216-s001.zip › healthcare-3023344-supplementary.pdf]

Supplementary Material

Supplementary File S1

S1. Keywords used for database search

(TITLE-ABS-KEY ("renal donation" OR "kidney donor*" OR "renal donor*" OR "kidney donation" OR "living kidney donor*") AND TITLE-ABS-KEY ("personal Growth" OR "quality of life" OR well-being OR "health-related quality of life" OR psycholog* OR anxiety OR depression OR health OR psychiatry OR adjustment OR emotion OR cope OR coping)) AND (LIMIT-TO (PUBYEAR , 2023) OR LIMIT-TO (PUBYEAR , 2022) OR LIMIT-TO (PUBYEAR , 2021) OR LIMIT-TO (PUBYEAR , 2020) OR LIMIT-TO (PUBYEAR , 2019) OR LIMIT-TO (PUBYEAR , 2018) OR LIMIT-TO (PUBYEAR , 2017) OR LIMIT-TO (PUBYEAR , 2016) OR LIMIT-TO (PUBYEAR , 2015) OR LIMIT-TO (PUBYEAR , 2014) OR LIMIT-TO (PUBYEAR , 2013) OR LIMIT-TO (PUBYEAR , 2012)) AND (LIMIT-TO (SUBJAREA , "MEDI") OR LIMIT-TO (SUBJAREA , "NURS") OR LIMIT-TO (SUBJAREA , "SOCI") OR LIMIT-TO (SUBJAREA , "MULT") OR LIMIT-TO (SUBJAREA , "PSYC") OR LIMIT-TO (SUBJAREA , "DECI") OR LIMIT-TO (SUBJAREA , "HEAL")) AND (LIMIT-TO (DOCTYPE , "ar")) AND (LIMIT-TO (LANGUAGE , "English"))

http://ezproxy.unibo.it/login?url=https://search.ebscohost.com/login.aspx?direct=true&db=pdh&db=psyh&db=ccm&bquery=(+(+%26quot%3brenal+donation%26quot%3b++OR++%26quot%3bkidney+donor*%26quot%3b++OR++%26quot%3brenal+donor*%26quot%3b++OR++%26quot%3bkidney+donation%26quot%3b++OR++%26quot%3bliving+kidney+donor*%26quot%3b+)+)+AND+(+(+%26quot%3bpersonal+Growth%26quot%3b++OR++%26quot%3bquality+of+life%26quot%3b++OR++well-being++OR++%26quot%3bhealth-related+quality+of+life%26quot%3b++OR++psycholog*++OR++anxiety++OR++depression++OR++health++OR++psychiatry++OR++adjustment++OR++cope++OR++coping+)+)&cli0=DT1&clv0=201201-202312&type=1&searchMode=Standard&site=ehost-live&scope=site

Scopus: 1395 records

CINAHL: 271 records

APA PsycInfo: 95 records
